# Young Adults’ Lived Experiences with Cancer-Related Cognitive Impairment: An Exploratory Qualitative Study

**DOI:** 10.3390/curroncol30060422

**Published:** 2023-06-09

**Authors:** Sitara Sharma, Jennifer Brunet

**Affiliations:** 1 School of Human Kinetics, University of Ottawa, Ottawa, ON K1N 6N5, Canada; 2 Cancer Therapeutic Program, Ottawa Hospital Research Institute, The Ottawa Hospital, Ottawa, ON K1H 8L6, Canada; 3 Institut du Savoir Montfort, Hôpital Montfort, Ottawa, ON K1N 6N5, Canada

**Keywords:** cognition, exercise, oncology, interviews

## Abstract

Cancer-related cognitive impairment (CRCI; e.g., disrupted memory, executive functioning, and information processing) affects many young adults, causing significant distress, reducing quality of life (QoL), and thwarting their ability to engage in professional, recreational, and social experiences. The purpose of this exploratory qualitative study was to investigate young adults’ lived experiences with CRCI, and any strategies (including physical activity) they use to self-manage this burdensome side effect. Sixteen young adults (M_age_ = 30.8 ± 6.0 years; 87.5% female; M_years since diagnosis_ = 3.2 ± 3) who reported clinically meaningful CRCI whilst completing an online survey were interviewed virtually. Four themes comprising 13 sub-themes were identified through an inductive thematic analysis: (1) *descriptions and interpretations of the CRCI phenomenon*, (2) *effects of CRCI on day-to-day and QoL*, (3) *cognitive–behavioural self-management strategies*, and (4) *recommendations for improving care*. Findings suggest CRCI is detrimental to young adults’ QoL and must be addressed more systematically in practice. Results also illuminate the promise of PA in coping with CRCI, but research is needed to confirm this association, test how and why this may occur, and determine optimal PA prescriptions for young adults to self-manage their CRCI.

## 1. Introduction

Annually, over one million young adults aged 18–39 years are diagnosed with cancer worldwide [1]. As their disease survival rate surpasses 80% [2], young adults are increasingly burdened with a host of physical and psychological sequelae that severely impair their daily functioning and quality of life (QoL) [3]. Cancer-related cognitive impairment (CRCI) is among the most common adverse effects reported by survivors across their lifespan [4], and is characterized by disturbances in mental processes related to thinking, reasoning, remembering, concentrating, learning, and processing information [5]. Since cognitive deficits often persist long after completion of treatment [6,7,8], many survivors experience significant psychological distress [9] and struggle with several emotional, interpersonal, and economic problems [10]. This is especially important to consider in a young adult population as unmanaged CRCI can disrupt their abilities to achieve major developmental milestones and establish functional roles in society [10]. However, published studies on CRCI have predominantly targeted middle-aged and older breast cancer survivors [5], and consequently, the extent and nature of young adults’ CRCI experiences remain poorly understood, resulting in inadequate management of CRCI in practice.

Another limitation of previous studies pertains to the methods used to assess CRCI, which have largely been quantitative in nature and thus provide limited insight into young adults’ lived experiences with this adverse effect. Given the clinical relevance of patient-reported outcome measures [11], self-report questionnaires are often used for cognitive assessment; the *European Organization for the Research and Treatment of Cancer Quality of Life Questionnaire* (EORTC QLQ-C30; [12]), *Functional Assessment of Cancer Therapy—Cognitive Function* (FACT-Cog; [13]), and *Cognitive Failures Questionnaire* [14] are some popular examples. However, whilst most have shown evidence of reliability and validity [15], there are critical conceptual issues related to the content of the self-report measures employed in oncology research. For instance, the EORTC QLQ-C30 is one of the most commonly used self-report instruments for assessing cognition in cancer survivors [16,17]; however, it was designed to measure QoL and only comprises two items related to cognition [12]. Additionally, comparison of extant questionnaires reveals substantial heterogeneity with respect to their cognitive focus (e.g., memory, attention), and measures geared towards young adults are lacking [15]. This not only constitutes a problem for comparing research findings, but using such questionnaires alone fails to yield a rounded understanding of which aspects of/issues with cognitive (dys)function are relevant and important to young adult cancer survivors. Correspondingly, qualitative methods are best suited to uncover their experiences with CRCI. Such methods are attracting increasing interest in oncology (e.g., [18,19]) because they allow for a thick, in-depth description of a phenomenon and the capturing of complex experiences that may not otherwise be explored [20]. Selamat et al. [21] synthesized the sparse corpus of qualitative research on CRCI with breast cancer survivors, concluding that survivors struggle to adjust to/manage cognitive impairments and face hardship on multiple levels (i.e., emotional, psychological, social, occupational). Although these findings *may* translate to young adults, ramifications likely vary according to life stage. Thus, to better understand young adults’ lived experiences with CRCI (including its specific burden on this group and potential self-management strategies), it is necessary to make use of qualitative methods.

Therapeutic options to prevent or treat CRCI remain elusive, but physical activity (PA) may help young adults *cope* with CRCI and/or *enhance* their cognition. The cognitive benefits of PA have been observed in several groups including healthy older adults [22], individuals with diseases of cognition (i.e., mild cognitive impairment, dementia) [23,24,25], and young persons with neurodevelopmental disorders (e.g., attention-deficit hyperactivity disorder) [26,27,28]. Studies have also shown promising results in cancer survivors. For instance, Galiano-Castillo et al. [29] reported improved performance on two neuropsychological tests assessing memory, executive functioning, and processing speed in middle-aged breast cancer survivors following a resistance PA intervention. Meanwhile, Gokal et al. [30] found improvements in self-reported cognition in middle-aged breast cancer survivors following a home-based aerobic PA intervention. Breast cancer survivors have also spoken in favour of PA as a behavioural strategy in a qualitative study [31], perceiving that it helped them reduce mental fatigue and improve mental clarity. Nevertheless, a major issue remains—evidence to support a link between PA and cognition in cancer survivors is mixed [17,32]. Therefore, qualitative inquiry into young adults’ PA beliefs and experiences as they relate to self-management of CRCI may shed light on the causes of such mixed findings and offer suggestions for creating future PA-based CRCI interventions and supports for this population.

### Current Study

The objectives of this qualitative study were twofold: (1) understand the lived experiences of young adults who report clinically meaningful CRCI after completing primary treatment for non-metastatic cancer, and (2) explore their use of strategies (including PA) to self-manage CRCI.

## 2. Materials and Methods

### 2.1. Design

This qualitative study was undertaken as part of a larger, mixed-methods observational study designed to explore how young adults experience and cope with CRCI after treatment, taking into consideration potential predisposing factors (i.e., medical, psychological), interventional strategies (i.e., PA), and outcomes (i.e., QoL) (quantitative results forthcoming). Both authors identify as women, and at the time of the study, they were a master’s student and an Associate Professor in the School of Human Kinetics at the University of Ottawa. The reporting herein complies with the *Consolidated Criteria for Reporting Qualitative Studies (COREQ)* checklist [33] (see Supplementary File S1).

### 2.2. Participants and Procedures

Following approval from the University of Ottawa Research Ethics Board (H-05-21-6889—REG-6889), young adults were recruited via social media advertisement, online postings on relevant organizations’ websites/newsletters, and word of mouth for the larger, mixed-methods study. Eligibility criteria were (1) cancer diagnosis between 15 to 39 years of age and currently aged 16 to 39 years, (2) completed primary treatment for non-metastatic cancer, (3) access to the Internet and audio–visual devices, and (4) ability to read, speak, and provide written informed consent in English. Young adults were ineligible to participate if they (1) had traumatic brain injury or concussion with residual symptoms (e.g., dizziness, headaches, loss of concentration) at the time of screening, (2) were actively taking selective serotonin reuptake inhibitor/serotonin norepinephrine reuptake inhibitor medication to treat a major mood disorder, and/or (3) received a diagnosis of a substance use disorder (e.g., alcohol, narcotics) by a medical professional within the past year. Participants were recruited from August 2021 to May 2022.

**Figure 1:**
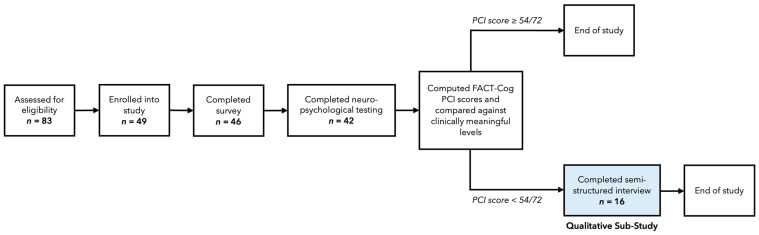
Overview of study flow.

An overview of study flow for the larger mixed-methods study is presented in Figure 1. In short, after providing informed consent, participants undertook two quantitative assessments: first, they completed an online survey with multiple questionnaires including the *Functional Assessment of Cancer Therapy—Cognitive Function* (FACT-Cog; [13]), followed by a brief battery of three web-based neuropsychological tests hosted on the *Inquisit 6 Web* platform. For this qualitative study, purposive sampling was used. Specifically, on a rolling basis, participants’ responses on the FACT-Cog were compared against clinically meaningful levels of cognitive impairment [34]; those who scored below 54 (out of a possible 72) on the 18-item *Perceived Cognitive Impairments* (PCI) subscale were invited via email to participate in a semi-structured interview. Sixteen of the 46 young adults enrolled in the larger study were invited, and all agreed to be interviewed (see Results for sample characteristics). At cessation of the larger study, participants were entered into a draw to win a CAD $100 gift card, with a total of three possible entries for each study component they began (i.e., survey, neuropsychological tests, interview).

### 2.3. Measures

#### 2.3.1. Sociodemographic and Medical Characteristics

To describe the sample, participants were asked to self-report their age, sex, gender identity, self-identified ethnicity, civil status, highest level of education attained, household income, employment status, medication, substance-use (if applicable), cancer type and stage, date of cancer diagnosis, and cancer treatment history. Participants also rated their perceived health on a 5-point Likert scale ranging from 1 (*excellent*) to 5 (*poor*) using a single item from the *36-Item Short Form Health Survey* [35].

#### 2.3.2. Self-Reported Cognitive Function

As mentioned above, the FACT-Cog (Version 3) [13] was used to assess self-reported cognitive function and identify participants for this study. The FACT-Cog is a 37-item measure designed specifically to assess cognitive impairment and its impact on QoL in cancer survivors over the past week. This questionnaire comprises four subscales (i.e., *PCI, Comments from Others, Perceived Cognitive Abilities,* and *Impact on QoL*) and responses are given using a 5-point Likert scale ranging from 0 (*never/not at all*) to 4 (*several times a day/very much*). While a total FACT-Cog score can be obtained by reverse-scoring negatively stated items and summing all items, only the 18-item PCI subscale score was used based on recommendations from scale developers (see the scoring document available at www.facit.org/measures/FACT-Cog, accessed on 15 May 2021) to select participants for interviews as described above. This specific subscale asks about difficulties related to forming thoughts, thinking, concentrating, remembering, communicating with others, reacting to situations, and both sustaining and shifting attention. Scores on the FACT-Cog (including PCI subscale scores) have been found to be reliable and valid, and this questionnaire has been used previously with various cancer populations [13,15].

#### 2.3.3. PA

The *Leisure-Time Exercise Questionnaire* (LTEQ; [36]) was used to assess PA levels and describe the sample to provide context for interpreting PA-related data. The first item asks participants how often they engage in mild-, moderate-, and strenuous-intensity PA for a minimum of 15 mins during their leisure time in a typical week. As recommendations for cancer survivors are to accumulate at least 150 mins of moderate-to-vigorous-intensity aerobic training per week for health benefits (e.g., www.cancer.org/healthy/eat-healthy-get-active/get-active/fitting-in-fitness.html, accessed on 30 April 2022), frequency scores for moderate and vigorous PA were multiplied by a corresponding metabolic equivalent for task (MET) value (i.e., moderate ×5; vigorous ×9) and summed to obtain a moderate-to-vigorous-intensity PA (MVPA) Leisure Score Index (LSI). Based on published LSI cut-points [37], participants were classified as either *active* (MVPA LSI ≥ 24) or *insufficiently active* (MVPA LSI < 24). LTEQ scores have demonstrated reliability and validity with accelerometer data [38], and this measure has been widely used in studies with adult cancer survivors [39].

#### 2.3.4. Interviews

The first author conducted individual semi-structured interviews with participants using an online platform (i.e., *Zoom*); these were audio-recorded and transcribed verbatim by SS within one week of the interview. On average, interviews lasted 69 mins (range = 42–91). The authors developed an interview guide with questions that focused on participants’ lived experiences with, and self-management strategies (including PA) for CRCI. To prompt participants to share their experiences, they were asked questions that centered on: (1) how they viewed their cognitive function and/or impairment, (2) what they perceived as predisposing factors to their CRCI, (3) how they felt these impairments impacted their QoL, (4) how they cope with CRCI, and (5) their thoughts on PA as a self-management strategy. Participants were encouraged to deviate from the interview questions to discuss experiences that had significant meaning to them, and all interviews ended with an opportunity for participants to make final comments and/or add additional pertinent information. Moreover, probes were used when responses lacked sufficient detail, depth, or clarity [40], and follow-up questions were used to further pursue central themes, elaborate on the context of answers, and explore the implications of what was said. Sample questions and probes used during the interview are presented in Table 1. During and immediately after the interviews, the first author took field notes to document any contextual information and observations necessary for conducting a quality analysis.

### 2.4. Interviewer

The interviewer (first author) was in her early 20s and had garnered research experience working with cancer survivors (including young adults) in the context of an exercise training/rehabilitation study. She had also received training in qualitative methods from the second author and as part of her graduate education. Her knowledge, skills, and experience made her ideally suited to develop rapport with participants and discuss their experiences with CRCI, as well as PA. Prior to the interviews, she pilot-tested the interview guide with a young adult cancer survivor who was selected purposively to help determine if questions were neutral, clear, flowed, and if it was feasible to conduct the interview in roughly one hour (to minimize participant burden). In doing so, she was also able to practice developing probes and follow-up questions. Data from this pilot interview were not included. However, based on feedback, she deleted one redundant question, re-arranged some for better flow, and made a note to begin each interview by defining “cognitive function” in lay terms to avoid confusion or misinterpretation of questions.

### 2.5. Sample Size

Given the lack of a definitive recommendation from experts for determining sample size in qualitative research, the criterion of data saturation [41] was used. That is, participants were approached and interviewed for this sub-study until no additional information appeared to be forthcoming; at this point, sampling was discontinued [42]. Saturation was achieved after the fifteenth interview; however, one additional interview was conducted with a participant who had expressed interest before recruitment was terminated, yielding a total sample size of 16.

### 2.6. Data Analysis

Interviews were transcribed using *NVivo Transcription* (Version 1.7.1). Transcripts were managed and analyzed in *Microsoft Word* (Version 2203) using inductive thematic analysis [43]. Analysis involved six steps: (1) familiarizing oneself with the data and generating initial codes, (2) systematically coding salient features of the raw data across all interviews that were relevant to the research objectives, (3) grouping similar codes to develop sub-themes, (4) reviewing and grouping similar sub-themes into main themes, (5) defining and naming themes and sub-themes to capture their essence, and (6) selecting compelling anonymized quotes from transcripts to illustrate each final theme/sub-theme and communicate participants’ experiences in a meaningful way. The first author was responsible for the formal analysis, and the second author provided input at each step; accordingly, codes, themes, and sub-themes were revised following joint reflection. Transcripts were not returned to participants for comments or corrections, and participants did not provide feedback on the findings.

### 2.7. Study Rigour

Several strategies were undertaken during this study to enhance the rigor and trustworthiness of qualitative data. First, the interview guide was pilot tested with a young adult cancer survivor. Second, open-ended questions were asked to allow participants to express what they felt was important and expand upon/alter responses as they wished. Third, the interviewer developed rapport with participants by being empathetic and attentive throughout, which is key to a constructive qualitative interview [44]. Fourth, an exhaustive, systematic, and reflective analysis of the data was conducted by the first author, and the second author acted as a “critical friend” [45] during the development and reporting of themes/sub-themes to encourage consideration of multiple and alternative interpretations of the data. Importantly, while interpreting data, both authors took time to acknowledge and reflect upon any preconceptions, personal experiences, and prior knowledge of the literature. Finally, detailed descriptions of the research process and analyses have been provided above in accordance with the COREQ checklist [33] to ensure explicit, transparent reporting, along with the quotations below to give participants voice.

## 3. Results

### 3.1. Sample

Participants were between 23 to 39 years of age (M = 30.8 ± 6.0) (see Table 2 for characteristics). Most were born female (*n* = 14; 87.5%;), self-identified as women (*n* = 14; 87.5%;) and White (*n* = 12; 75%), single (*n* = 8; 50%), had completed post-secondary education (*n* = 15; 93.8%), were either working or transitioning into work (*n* = 10; 62.5%), and had an annual household income <CAD $100,000 (*n* = 12; 75%). In terms of medical characteristics, participants were between 15 to 38 years of age at diagnosis (M = 27.6 ± 7.9), and their time since diagnosis ranged from 0 to 10 years (M = 3.2 ± 3). There was diversity in cancer stage, type, and treatments reported, but most were diagnosed with stage II cancer (*n* = 7; 43.8%), a hematological cancer (25%; *n* = 4), and received surgery as primary treatment (*n* = 13; 81.3%). Also, participants largely perceived their overall health as “good to very good” (*n* = 10; 62.5%). Previous concussion(s) and cannabis use within the past month was reported by two (12.5%) and seven (43.8%) participants, respectively. Also, participants were *insufficiently active* on average, based on their self-reported MVPA (M = 19 ± 12.7; range = 0–46); however, seven (43.8%) had a MVPA LSI score ≥ 24 (i.e., the established cut-point [37] for being classified as “active”). For a better understanding of the sample, the profiles of participants who were interviewed are noted in Table 3.

### 3.2. Themes

**Figure 2:**
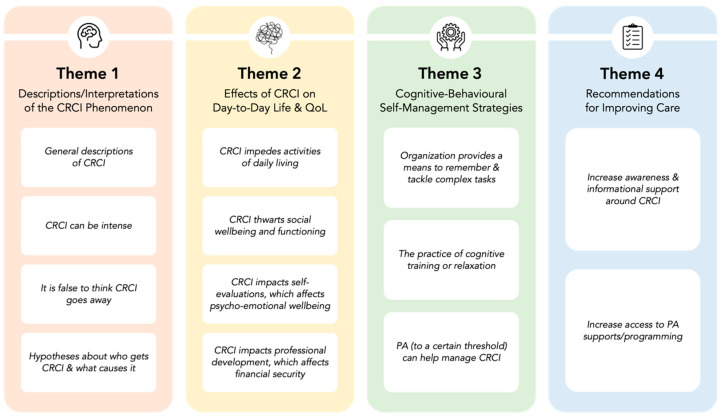
Main themes and sub-themes encompassing young adults’ lived experiences with CRCI.

As displayed in Figure 2, four themes comprising 13 sub-themes were developed based on the data: (1) *descriptions and interpretations of the CRCI phenomenon*, (2) *effects of CRCI on day-to-day and QoL*, (3) *cognitive–behavioural self-management strategies*, and (4) *recommendations for improving care*. Each theme is presented below, supported by quotations from individuals identified by pseudonyms. Of note, in the quotations, […] indicates that text was omitted to enhance clarity.

#### 3.2.1. Theme 1: Descriptions and Interpretations of the CRCI Phenomenon

The first theme captures participants’ thoughts about the origins, evolution, and meaning of CRCI following cancer treatment. These were organized into four sub-themes: *general descriptions of CRCI, CRCI can be intense*, *it is false to think CRCI always goes away*, and *hypotheses about who gets CRCI and what causes it*.

The *general descriptions of CRCI* sub-theme illustrates the meanings that participants ascribed to their cognitive impairment, which was painted out to be “fog”-like (Nina), a “constant cloud” (Jaime), and a “black hole” (Priya). According to Sydney, CRCI makes “everything [feel] like it’s been muted a bit… like… when you’re sick and your brain’s just not moving quite at [the right] speed”. She went on to say, “I feel like that all the time, but I’m not sick anymore”. Participants illuminated troubles with their memory, word recollection, concentration, and ability to both process and learn information. For Jack and Mia respectively, these deficits added “a layer of difficulty” to everything and made it feel as if she “can’t trust [her] brain”.

As reflected within the *CRCI can be intense* sub-theme, participants’ cognitive impairment often presented frequently and with considerable severity. Lauren remarked, “It’s hard to say how frequently I have *actual* issues, but… it comes to my attention that I am having this problem… at least once or twice a week”. Others affirmed struggling with cognitive impairment even more often; that is, either “multiple times a week” (Peyton) or “pretty much every day” (Nina). CRCI was such a constant for Jack that he explained, “I basically build the way that I interact around [CRCI]”. When asked to describe their CRCI severity on a scale from 0 to 10, ratings ranged from “two” (Erica) to “severely… 10” (Ivy), although most felt it landed right in the middle of the scale. On average, as Peyton explained, “it bothers me, obviously, but… I can still live my life around it”. Further, CRCI severity was described to be fluid, such that “some days might be less [severe] than others” (Layla), and that “it’s definitely worse [on] the days that [they] do more” (Sarah).

The *it is false to think CRCI goes away* sub-theme encompasses an unfortunate reality. Cognitive impairment was most pronounced during primary treatment and immediately after it had ended. Participants described a “rapid drop” (Jack) in their cognitive function during treatment that was “consuming” (Layla). Indeed, in recalling her experience during this time, Emma said, “I don’t think I was functioning at all cognitively”. Whilst unsurprising as participants all self-reported clinically meaningful CRCI, cognitive difficulties were typically worse during and immediately after treatment, but many continued to struggle post-treatment. Cole explained that his CRCI got “progressively worse,” while others characterized CRCI as dynamic. For instance, Mia explained that her cognitive function changed “in waves,” wherein it vacillated between improving and worsening depending on adjustments to medication. Others also noted that their cognitive function continuously changed and that “it’s been better than during treatment… but it’s definitely not a huge improvement” (Emma). Importantly, while slight-to-moderate improvements in cognitive function were discussed, participants largely credited these to “work[ing] really hard” (Layla) to adjust to and self-manage their CRCI because they accepted cognitive impairment as a permanent side effect that they needed to get used to. As Peyton exemplified, “I don’t know… if [my cognitive function is] getting better, or if I’m just getting… used to living with how my brain works”.

Finally, the *hypotheses about who gets CRCI and what causes it sub-*theme reflects that participants largely attributed CRCI to treatments received; since individuals diagnosed with different cancers (i.e., type, stage) receive common treatments (e.g., chemotherapy, radiation therapy, surgery, medications), participants felt that CRCI could affect *anybody* receiving treatment. Highlighting this, Emma said, “I think treatment…” when speaking to the causes. She then added, “I feel like its [affected] everybody that I’ve talked to”. Likewise, Lauren said, “My guess is that people experience [CRCI] with cancer treatment in general. I don’t know if that has to do with the fact that you’re given like, so many drugs…and all that just messes with your brain… I feel like in general, cancer patients… have some sort of cognitive issues related to treatment”.

#### 3.2.2. Theme 2: Effects of CRCI on Day-to-Day Life and QoL

The second theme demonstrates that participants explicitly linked CRCI to QoL and reveals its tremendous, multidimensional burden. Specifically, participants noted that when cognitive troubles manifested, their physical, social, psycho-emotional, and professional wellbeing and functioning were adversely impacted. Consequences were grouped into four sub-themes: *CRCI impedes activities of daily living, CRCI thwarts social wellbeing and functioning, CRCI impacts self-evaluations which affects psycho-emotional wellbeing, and CRCI obstructs professional development which affects financial security*.

The *CRCI impedes activities of daily living* sub-theme captures how CRCI thwarts one’s ability to undertake instrumental activities of daily living (IADL); that is, key life tasks needed to live independently and maintain health. Participants described basic tasks such as cooking and housekeeping as challenging because “everything takes more focus, more work” (Sarah) and because they would get easily distracted. For instance, Peyton said, “baking… cooking… laundry… it just takes longer to do stuff and [requires] being more thorough because I have to like, go back and make sure, or like, re-read or that kind of stuff”. Due to the extra time and effort required to complete such tasks, participants often had less time for engaging in PA; as Taylor conveyed, “Stuff takes longer for me… [so] I don’t leave enough time for my walks”. Additionally, some neglected basic self-care due to their cognitive struggles, saying “this sounds so gross, but I’d forget to brush my teeth, or I would forget to eat breakfast or something like that” (Erica). Participants also mentioned difficulties with upholding personal values such as being punctual. Sarah said, “I was never late for anything before… Now I’m late for everything and I hate it. It’s like there’s not enough time in a day for me to get through anything. I just seem like I’m failing a lot”. Moreover, driving was discussed as another common IADL affected by CRCI. Particularly due to difficulties with focusing and processing situations, participants limited or stopped driving out of apprehension for threatening the physical safety of oneself and others. Priya remarked, “I’ve been really nervous about… driving just because I feel like my reaction time is kind of slow… like if someone ran out in front of my car or turned suddenly, would I be able to react as fast as I could before?” Mirroring this hesitation, Sarah said, “There has been a couple times driving where… I just have come home because I know that I shouldn’t be out there because I can’t focus enough. Or I’ve had a close call or something, right? Where I’m like, ‘Hey… I’m not here.’ So there ha[ve] been those days where I just shut it down”.

The *CRCI thwarts social wellbeing and functioning* sub-theme reflects that CRCI strained relationships with others (e.g., romantic, familial, friendships) as it often caused communication struggles and left individuals feeling misunderstood. For example, Sydney said, “I do need more time to process… [than my] very quick-thinking partner… that alone is frustrating. I mean, that’s basically the crux of like all of our communication problems”. Further, Mia mentioned that she “ha[s] a father who… doesn’t understand being forgetful. He doesn’t understand that [CRCI] is something that I’m dealing with so he’ll get upset, saying that I don’t remember something because I don’t want to do it”. Lack of understanding and negative comments from others were also discussed by Lauren, who explained, “I would get very hurt when people would tell me… ‘Oh, you don’t remember this?’… my memory is not as great… it’s hard for the people in my life to understand that”. Moreover, participants described feeling like a “bad friend” (Taylor) because CRCI made it difficult for them to be as thoughtful or to recall memories and details about loved ones’ lives. Collectively, cognitive difficulties created “an internal barrier” (Emma) that inhibited social engagement and led to self-isolation for participants. For instance, Sarah said, “Because I can’t articulate how I feel, I just avoid… some of my family…. They don’t understand”. Similarly, Nina expressed, “Sometimes when I can’t put a sentence together, I feel really ridiculous and then it kind of makes you not want to talk to people because you feel like… they’re probably thinking, ‘Oh my gosh, what’s going on with her?’ So… maybe I am a little bit more isolating myself”.

The *CRCI impacts self-evaluations which affects psycho-emotional wellbeing* sub-theme captures the heavy inward struggles and distress that CRCI causes. Participants perceived themselves as “a failure” (Emma), a “damaged version” of themselves (Taylor), and “like a shell of… what [they] once thought [they were]” (Peyton) due to their CRCI. Importantly, their damaged self-concept and identity often stemmed from feeling less intelligent than prior to their cancer experience and gave rise to many negative emotions and thoughts. Sarah shared, “It makes you insecure… You don’t recognize your brain, right? All the things that you grew up learning how to study and knowing how to do… they don’t work anymore… It’s very unnerving… not being who you were and not being as smart as you were”. Correspondingly, Layla expressed, “My memory was quite sharp… I always got high grades. So, it was really, really disappointing. I think [CRCI] took a lot of my identity. I lost a lot of my confidence in myself and developed a lot of imposter syndrome… Initially, [CRCI] was really, really upsetting… I went home and cried every day… it was just so hard and frustrating”. Paradoxically, negative emotions and thoughts further fueled cognitive troubles, suggesting that the connection between CRCI and psychological distress is bidirectional, or as Taylor put it, a “vicious cycle”. Jack explained “I think it happens in both directions. I think if I’m having issues with my cognition, I feel depressed and slow and low. And conversely if I’m having anxiety… I’m so hyper-focused on those things that I can’t pay attention well, I get distracted, I can’t remember what’s going on… I think they feed all on each other like writhing massive snakes”.

Finally, the *CRCI obstructs professional development which affects financial security* sub-theme encapsulates the toll CRCI takes on work/school performance and motivation to pursue vocational opportunities. Nina exemplified this when she said, “I did decide to step back a little bit from work because I felt like I…couldn’t function [cognitively]… I can’t make mistakes in my work… I’ve cut down my hours. So yeah, it affected me financially… And it’s frustrating because, like, if I was in my 50s or 60s, I’d probably be retired, and I wouldn’t have to work. It wouldn’t matter, you know? But it matters right now for me because I’m so young… I probably have to work another twenty years”. As Nina alluded to, young adults bear many financial responsibilities, making this consequence of CRCI especially taxing for them. Moreover, based on the survey data collected, four (25%) participants were currently unemployed or on medical leave, and they described CRCI as a barrier to return to work or school. Cole explained, “It’s been very hard to be able to consistently stay with like a full-time job… There’s so much to learn… There’s so many mistakes that you can make in a day… It just seems that every attempt has been futile for me”. Others postponed return to work or ceased their job searches out of fear or discouragement that it would “take [them] longer to finish tasks” (Eva) and ultimately, that they would not “bring value to the team” (Peyton). As Mia said, “It’s not fair to a new employer for me to go there and have all of this confusion and everything until I’m all sorted out”.

#### 3.2.3. Theme 3: Cognitive–Behavioural Self-Management Strategies

The third theme reflects the cognitive–behavioural self-management strategies participants used to self-manage their CRCI. The strategies described herein were used to help participants cope or to improve their ability to remember, focus, and tackle complex tasks. Of note, none of the strategies were unanimously used, highlighting the personal nature of dealing with CRCI and the potential need for tailored interventions. Despite the diversity in use, strategies are captured within three non-exclusive sub-themes: *organization provides a means to remember and tackle complex tasks, the practice of cognitive training or relaxation, and PA (to a certain threshold) can help self-manage CRCI*.

The *organization provides a means to remember and tackle complex tasks* sub-theme captures the various organizational methods and tools participants used to help them self-manage their CRCI (in some cases), specifically by helping to jog their memory and reserve their cognitive energy. To help manage troubles with memory, participants described “stick[ing] to a routine” (Mia), scheduling “everything in a calendar” with constant reminder alerts (Sarah), “writ[ing] everything down” (Emma), and essentially, as Jaime explained, “putting [stuff] somewhere that’s not inside my head”. However, for others, “calendars and all that stuff… just doesn’t work” (Cole) because ironically, they were often forgotten or misplaced. For instance, Jack said, “I try and make lists and then a few days later, I forget that I made a list. And then a few months later, I’m going through, like erasing iPhone notes and going, ‘Oh, I was supposed to do this or that’… So, I do make lists, but I’m not successful at using them”. Beyond routines, scheduling, and notes/lists, participants mentioned organizing their days intentionally to undertake certain tasks when their cognitive function was at its “best”. For example, Layla mentioned, “I know that I have better [cognitive] function in the morning. So, [I try] putting more complex things in the beginning of my day versus trying to do them in the afternoon because I’m exhausted and I… don’t have the capacity as much”. Overall, organization was used as a tool to help manage (not improve) poor memory and attention.

The *practice of cognitive training or relaxation* sub-theme captures techniques that participants used to help them better remember and focus. On one hand, some participants integrated different cognitively demanding games/activities into their routines to “get [their] brain[s] working” (Taylor). Erica touched on many of them when saying that “listening to podcasts and that kind of thing... keep[s] my brain working. One thing that I got really into during treatment… is sudoku puzzles… just remembering those little things I thought was really good practice… It made me focus and… utilize my short-term memory”. Similarly, Taylor said, “I started to play a lot of solitaire on my phone… sometimes I feel like it helps me to like, get my brain working”. Other examples Taylor gave included not “turn[ing] on [TV] subtitles so that [she had] to focus more” and “try[ing] to use… scientific papers that are in English [when conducting research for university] so that it’s harder” (as English is not her first language). Conversely, others wanted to calm their brains through cognitive relaxation techniques which helped them enhance focus and memory while also reducing stress. For instance, Erica cited, “Meditation is like my number one [strategy]… it is amazing. It has all these benefits… I find my memory is better,” and Ivy described that a simple five-minute guided meditation session helped her go “from being, like, very frazzled and fe[eling] like [her] head [is] being pulled in a million different directions to… just like, all of a sudden [feeling that things were] manageable… it helps put the pieces in order”.

Finally, the *PA (to a certain threshold) can help manage CRCI* sub-theme captures that PA was used for various purposes, including allowing participants to better concentrate, remember, and “not be stuck in that… weird foggy state as much” (Priya). As shared by Jack and Erica, respectively, “I can [focus] whenever I come back from exercise, I get so much done… I’m just more successful,” and “I find that the days that I’ve gone to the gym the day before… I feel like my memory is better… I feel more alert”. Emma found that PA “definitely helped [her] brain” and made it easier for her to tackle tasks that were otherwise difficult. Furthermore, Ivy mentioned that the act of counting strokes and focusing on her breath while swimming (i.e., incorporating mindfulness) helped calm her brain and thus provided some relief from her cognitive struggles, tying into the above sub-theme. She said, “For me, [when I swim laps] … the counting of strokes and breath… it’s just so meditative… [I feel] mental effects and benefits”. Critically, the key was to engage in PA that was not too intense or undertaken too often to avoid overexertion, as this could lead to cognitive fatigue. For instance, Emma mentioned, “If I push myself too hard [during PA], I’m just done. Like everything—physically, emotionally, cognitively, it’s just like total body shutdown… I am trying to learn the window where it feels good, when it’s not too much”. Similarly, Layla explained, “I started working out with a personal trainer in the summer… if I worked out too many days… the physical fatigue contribute[d] to increased brain fog… I tried working out… 3–4 times a week, and I had to cut it back to twice a week because… it was too much physically, and it really impacted my brain”. However, Layla went on to say, “I think other than the overexertion and getting to the fatigue point… I feel good in myself… [PA] helps with… your mental health, and then that translates into having better cognitive function”. Although PA was not something used by all to self-manage CRCI, even those *not* using PA as a strategy believed the benefits of PA likely extend to cognitive function. As Sydney conveyed, “PA clears your mind, so I can certainly… see the link [between PA and cognitive function]”. However, PA was not something that everyone knew how to engage in, which was clear when Sydney went on to say “I don’t know exactly… which type of PA… or if there’s kind of a strategy of ‘you should be doing *these* five things’ and ‘you work them in *this* order’ or whatever… But… I can see the reason that they would be linked for sure”.

#### 3.2.4. Theme 4: Recommendations for Improving Care

Participants unanimously described feeling ill-informed about CRCI and did not believe they “ha[d] the tools necessary in place that would have helped” (Cole) to navigate this challenging side effect. As such, they provided recommendations for improving care, which are captured within this final theme. Suggestions for successful survivorship were grouped into two sub-themes: *increased informational support around CRCI* and *greater access to PA supports/programming*.

The *increased informational support around CRCI* sub-theme captures participants’ desires for more systematic awareness and information around CRCI. Sarah expressed, “I think the more awareness we have that this is a real thing that all cancer patients go through, we won’t feel so alienated by it”. Likewise, Eva said, “Difficulty focusing… memory issues… [health professionals] don’t tell you about these things and you don’t expect it, and then it actually happens to you. You feel like something’s wrong with you when it’s not. So, I think it’s important to discuss that with you”. Many echoed that they felt they were left to understand and heal with cognitive impairment via “trial and error” (Peyton); thus, Mia remarked, “It would have been better if [health professionals] said, ‘*These* are some of the symptoms that you may have. If you run into these, *these* are some of the coping strategies you can deal with,’ instead of leaving it up to me to go onto these Facebook groups”. By the same token, Layla remarked “I think preparing people in advance for [CRCI] would be helpful… Medical professionals need… to help with cognitive strategies… To be like, ‘Yeah, so you may just have to write things down more.’ Like I know…it should be intuitive, but it wasn’t in that moment. So, like… ‘Write things down more,’ ‘chunk things up in your day’… ‘have [complex] things in the morning.’ Like those things would have been really really helpful tips because you’re dealing with something that you’ve never thought about or you never had to do”.

The *greater access to PA supports/programming* sub-theme reflects participants’ overwhelming desire for PA support during and following cancer treatment (both for CRCI self-management purposes and general health reasons) as it was often not presented as an option or was inaccessible to them. In relation to CRCI, Taylor explained, “[My friend] was prescribed physiotherapy during her treatment… Her cognitive function, I would say, is better than mine… She always had to do workouts during treatment, but when I was in treatment, they were like ‘Just stay in bed and just rest.’ And they told her ‘You should move. You should go out of the house at least once every day.’ I feel like if my doctors would have told me the same… that I would definitely have increased my cognitive function or made it less worse”. More broadly, participants mentioned that they would have liked more dialogue and information around the benefits of PA and how to engage in it safely and effectively. For instance, Priya said, “It would have been really helpful if the doctor had more of a conversation about PA… Like providing some kind of tips or even directing people to some resources”. In parallel, Layla said, “Some sort of… graphic info sheet that had either organizations you could access… as well as information on PA, the importance of it, how much PA cancer patients should be doing, or what activities they should be partaking in versus what’s contraindicated… would be helpful… I think… when people are [doing] really, really poorly and they’re trying to incorporate exercise, they need to know distance and frequency and timing and that kind of stuff to help”. Furthermore, participants believed that help from PA professionals (e.g., kinesiologists) in creating detailed, individualized PA plans would be beneficial in setting them (and future young adults) up for success following cancer treatment. As Sydney mentioned, “An actual recommendation to say, ‘We’d like to have you work with somebody to set up what would be a good physical exercise plan for you’…would go pretty far”.

## 4. Discussion

The purpose of this qualitative study was to better understand young adults’ lived experiences with, and self-management strategies for CRCI after completing primary cancer treatment. Overall, results encapsulated by Themes 1 and 2 extend previous research describing the adverse impacts of CRCI on cancer survivors, both in general, and for young adults specifically. Findings captured within Theme 3 support the continued investigation into several cognitive–behavioural strategies (i.e., organization, cognitive training or relaxation, PA) that may help young adults manage this burdensome side effect and suit different preferences. Finally, Theme 4 emphasizes the significance of acknowledging CRCI in research and practice, and the critical need for greater support.

### 4.1. CRCI Is Consequential for Young Adults

Results revealed the multidimensional consequences that clinically meaningful CRCI has for young adults’ QoL, including diminished daily functioning and independence, social and psycho-emotional well-being, professional capabilities, and financial health. This aligns with previous research involving adolescent and young adult cancer survivors [46,47,48,49] and breast cancer survivors [50,51,52,53]. Additionally, results suggest a bidirectional relationship (or “vicious cycle”) between CRCI and psychological outcomes (e.g., depressive symptoms, anxiety, perceived stress). Whilst shown to be associated [8,9,54,55,56,57,58], the lack of longitudinal studies (with repeated measures) that test the two causal directions between CRCI and psychological distress limits confirmation of a bidirectional association, and research exploring underpinning mechanisms (e.g., psychosocial, physiological) is also scarce; these gaps warrant future investigation.

Further, results illuminated that clinically meaningful CRCI affects young adults’ views of themselves. This is not surprising as cancer influences young adults’ self-evaluations and identify [59]. Current findings add that CRCI can leave young adults feeling less intelligent and threaten their sense of self, which can in turn hinder their vocational aspirations and success [60]. Seeing as young adults constitute a substantial proportion of the workforce [61], such feelings can represent a larger societal issue. Therefore, considering the pressures and difficulties participants described around resuming vocational pursuits after treatment, along with evidence that CRCI can impede occupational re-integration, reduce work capability, and cause job loss [47,48,49,50,52,62], it is crucial to help young adult cancer survivors maintain positive views of themselves. Investigating reasons behind such views will aid in identifying risk and protective factors to target when designing supportive care for young adults with clinically meaningful CRCI.

Finally, findings show discordance between young adults’ lived experiences and current CRCI measures which lack appraisal of a seemingly important construct—sense of self. This indicates a need to expand CRCI measures designed to capture its impact (e.g., FACT-Cog [13]), and involving young adults in their development/refinement may help increase relevancy. Relatedly, conceptual definitions of CRCI are simplistic and researcher-developed, but results suggest they can be modified to better capture the nuanced meanings young adults ascribe to their cognitive impairment. To do so and escape the limitations of postpositivist epistemologies, qualitative studies are necessary; arguably, these could also be used to build a theoretical framework of CRCI for young adults to support future research and practice.

### 4.2. Self-Managing CRCI

Similar to research with breast cancer survivors [21], results revealed that young adults with clinically meaningful CRCI struggle to understand, adjust to, and cope with their CRCI given a lack of informational support or resources; this forced many to explore compensatory cognitive–behavioural strategies on their own. As with previous findings [31,50], participants relied heavily on *external* organizational strategies (e.g., to-do lists, scheduling, setting alerts) as memory aids; however, others forgot about or misplaced the very tools they relied on to help them better remember, suggesting such strategies may not suit everyone. Instead, young adults may need to be taught how to use *internal* strategies (e.g., rehearsing/repeating/visualizing information, creating mnemonics/rhymes) to facilitate deeper information association and processing and thus help compensate for memory difficulties, as suggested in research with other populations (e.g., mild cognitive impairment, traumatic brain injury, stroke) [63]. Identifying which strategies work “best” for whom and under what circumstances would help inform decision making.

Also consistent with past studies [50,51,64,65], cognitive training (i.e., “exercising the brain” through mentally challenging games/tasks [e.g., solitaire, sudoku]) was used to manage troubles related to memory and focus. This is unsurprising given that “brain training” has received growing attention in media and research, and several mobile applications claiming to maintain/increase cognitive skills (e.g., *Lumosity, Elevate, CogniFit*) are available [66]. Systematic reviews of CRCI studies with adults [67] and breast cancer survivors [68] have identified cognitive training as an effective *rehabilitation* strategy for strengthening specific cognitive domains. Pilot data from adolescent and young adult cancer survivors [69] suggests it may also be a feasible, possibly beneficial CRCI *prehabilitation* tool; however, evidence is needed to confirm the effectiveness of cognitive training in young adults. Likewise, cognitive relaxation techniques were seen as beneficial to “calm the brain” (and in turn, help improve memory), supporting prior research (e.g., [70]). Whilst meditation was the only technique explicitly mentioned by participants, several others (e.g., mindfulness-based stress reduction [71,72], biofeedback [73], imagery [74]) have been investigated as CRCI interventions in breast and mixed cancer groups and elicited improvements in perceived cognition. As with cognitive training, research is needed to generate evidence on the effectiveness of cognitive relaxation techniques in young adults. Exploring underpinning mechanisms may also help understand how to target specific cognitive domains.

Furthermore, aligning with evidence from adolescent and young adult cancer survivors [75,76] and breast cancer survivors [31], findings emphasize that PA may be an effective CRCI self-management strategy for young adults. Indeed, feeling more focused, alert, and able to remember following bouts of PA were cited as cognitive benefits, and even those who did *not* use PA as an explicit strategy believed it *could* help manage CRCI. This suggests young adults might be willing to engage in PA for their cognitive and mental well-being, and thus, PA-based interventions for ameliorating CRCI should be developed and evaluated. However, not all PA interventions have conferred cognitive benefits (e.g., [77,78]), and some participants herein said not all PA was “good”. To inform effective PA intervention design for CRCI, several questions remain, including: *how much PA is needed to induce cognitive benefits, what types/combinations are most beneficial, and how long do the effects of PA last on cognition?* Regarding the latter, as previously suggested [79], the potential cognitive benefits of PA may be more acute than long-lasting. Thus, researchers may wish to investigate the effects of daily PA on young adults’ cognition using *Ecological Momentary Assessment* methodology [80] to see if cognitive benefits from PA are indeed acute and/or sustainable.

Interestingly, some participants enjoyed *mindful* PA (i.e., PA involving a heightened sense of attention; e.g., Qigong, yoga), whereas others alluded to benefits following gym-based resistance training; if well-practiced, the latter could be considered a form of *mindless* PA (i.e., PA that allows automaticity to take over). That said, the relative effects of mindful versus mindless PA remain unclear. Diamond and Ling [81] proposed that PA with a cognitive load (i.e., mental effort) may lead to “better” executive functioning than mindless PA; however, this hypothesis has been criticized due to a lack of empirical evidence [82]. Also, the current findings suggest that (a) individuals may prefer one type over the other, and (b) there could be cognitive benefits to both that go beyond executive functioning. Whilst questions on this topic were not asked in this study, findings suggest it may be fruitful to compare the effects of mindful and mindless PA on various cognitive domains in a larger sample of young adult cancer survivors and across different contexts.

While the theory that PA involving a cognitive load is superior to PA with a lesser cognitive component requires substantiation, participants herein used cognitive training or relaxation *and* PA as forms of self-management. This raises the question: *Can additive cognitive effects be experienced by combining these interventions?* Although such work is lacking in oncology, a recent systematic review concluded that PA programs enriched with mental challenges (e.g., exergaming, tai chi, dance) helped improve cognition in older adults with/without mild cognitive impairment [83]. Studies conducted across different groups further support the notion that combining cognitive training with PA may be more beneficial for the brain than PA alone (e.g., [84,85]); it is worth exploring if similar results map onto the young adult cancer population. Conversely, some have examined the effects of mindfulness-based interventions *compared to* PA on CRCI and reported cognitive improvements in both groups (e.g., [86]), but little is known about the impact of combining cognitive relaxation techniques with PA—another area requiring future research.

Nonetheless, certain barriers may stand in the way of young adults’ PA participation. This study suggests CRCI can serve as a barrier due to the extra time and effort required to complete essential daily tasks when struggling with this adverse effect. As evidence grows in support of the positive effect(s) of PA on cognition in cancer survivors (e.g., [75,76,87,88,89,90,91,92,93,94,95,96,97,98,99,100,101,102]), more messaging is needed to inform young adults about its benefits as a way to help them increase motivation and perhaps overcome barriers. Relatedly, drawing on the *Health Belief Model* [103] for predicting and explaining health behaviours, researchers should aim to identify and understand young adult cancer survivors’ perceived benefits, barriers, and self-efficacy in regards to engaging in PA for their brain health; this may help guide the creation of preliminary PA-based CRCI self-management for this group.

Finally, it is worth cautioning young adult cancer survivors that engaging in “too much” PA may compromise their cognition, as participants believed it caused or exacerbated mental fatigue. Drawing on sport psychology models (e.g., *Individual Zones for Optimal Functioning Model* [104,105]) that posit athletic performance is optimal up until/within a certain individualized zone of arousal, there may be an individualized threshold for which PA induces optimal cognitive effects based on one’s level of mental fatigue. This may require that healthcare practitioners encourage young adults to actively monitor themselves over time to determine their mental “sweet spot,” and engage in PA at this point. Moreover, building off the emerging practice of “personalized medicine” [106] which aims to tailor care based on individual differences (e.g., genes, environment), it would be valuable to study how to match young adults to the right PA parameters (e.g., frequency, intensity) so they may feel cognitive benefits and avoid mental fatigue.

### 4.3. Limitations and Future Directions

Although this study makes important contributions, it is not without limitations. First, the sample was predominantly comprised of women who were White, had completed post-secondary education, and had an annual household income above CAD $100,000; thus, results from this sample may not be transferable to all cancer survivors. Researchers should aim to adopt more diverse recruitment strategies that allow for maximum sample variation. Second, results predominantly captured the experiences of those who underwent chemotherapy exposure. To better target and understand the CRCI experiences of a wider young adult population, researchers should aim to recruit young adults who undergo anti-cancer treatments other than chemotherapy. Third, this sample had a large range in time since diagnosis (0–10 years); although perspectives were consistent across participants in this study, they may differ across others with shorter versus longer times since diagnosis. Researchers may wish to consider splitting analyses by time groups (e.g., one, five, ten years) and employing longitudinal designs to better understand the evolution of CRCI. Likewise, variations that stem from other personal (e.g., age, life events) and sociocultural (e.g., ethnicity, sociodemographic status) experiences may influence how these themes present across other individuals and require future purposive sampling and cohort research. Fourth, most (62.5%) participants perceived their overall health as “good to very good” and the sample was generally classified as “active” based on their self-reported PA; future studies are needed to confirm if the themes reported herein are similar or differ from those with young adults who do *not* view themselves as healthy or active. Fifth, there are inherent limitations to using self-report measures (e.g., social desirability, recall bias) whereby participants may have under- or over-estimated their perceived cognitive impairments and thus influenced who was invited for an interview. Also, since inclusion criteria for this study included scoring below a certain threshold on the FACT-Cog PCI subscale, if only one cognitive dimension (e.g., memory) was affected, it could have been masked with the overall PCI score. Sixth, the sample may be biased towards those with greater computer literacy and/or ability to spend time online given the methods used, suggesting the need to enhance accessibility of future virtual studies for those with CRCI. Finally, there is inherent subjectivity in thematic analysis wherein the researchers’ own biases and assumptions could have affected identification and interpretation of the themes/subthemes presented, although several steps were undertaken to mitigate this risk (see Study Rigour above).

## 5. Conclusions

The results show that young adults with clinically meaningful CRCI face deleterious consequences for their daily and overall QoL and suggest they experience CRCI differently than older cancer cohorts (i.e., those whom current definitions of CRCI are currently based on). Moreover, the findings reveal that young adults use several cognitive–behavioural strategies including organization, cognitive training and relaxation, and PA, highlighting that “one size may not fit all” when it comes to managing CRCI. This provides support for continuing to investigate how different forms of self-management (in isolation and in combination) may elicit cognitive benefits to appeal to the preferences of a wider range of survivors. Findings from this study also add to the growing body of research exploring links between cognitive function and PA and suggest more high-quality experimental research is needed to test the putative mechanisms underlying potential benefits of PA as well as optimal PA dosages/contexts. Last, this study lays important groundwork for creating CRCI-self-management supports for this underrepresented population and reinforces that young adults would benefit from more systematic awareness, assessment, and monitoring of CRCI in healthcare.

## Figures and Tables

**Table 1:** Example questions and probes from the semi-structured interview guide.

Question Categories	Example Questions and Probes
How participants viewed their cognitive function and/or difficulties.	Can you describe your current cognitive function? [Probe] What specific cognitive difficulties or impairments (e.g., impaired memory, attention, ability to process information, etc.) do you experience? Compared to before you were diagnosed, how do you think your cognitive function has changed over the course of your cancer journey? [Probes] After diagnosis? During treatment? Immediately after treatment? What about as time went on after treatment?
What they perceived as predisposing factors to their CRCI.	Do you think that being diagnosed when you were ___ years old and now being where you are in life influence how you experience your cognitive difficulties or impairments? How so? Do you think the cognitive difficulties or impairments you are experiencing are due to your specific cancer (i.e., ____) and/or the medications you have received, namely _____? Why/why not?
How they felt these impairments have impacted their QoL.	Can you tell me how the cognitive difficulties or impairments you mentioned have affected your emotional and/or psychological wellbeing? [Probe] Has this affected the way you see yourself? How?
How they cope with CRCI.	What strategies do you use to manage your cognitive difficulties or impairments? Why these? [Probe] Was/is physical activity one of such strategies? Why so? Why not?
Participants’ thoughts on PA as a self-management strategy.	Do you believe PA can improve your cognitive function? Why/why not? [Probes] Do you feel that PA helps improve your memory? Attention? Processing speed? Any other specific cognitive domains? Why or why not? Has anyone ever recommended that you engage in physical activity to improve your cognitive function? If so, who? What did they say?

*Notes.* CRCI = cancer-related cognitive impairment; QoL = quality of life; PA = physical activity.

**Table 2:** Sociodemographic and medical characteristics for interviewed participants (*n* = 16).

Variables	Values
**Sociodemographic Characteristics**	
Current Age (M Years ± SD; Range)	30.8 ± 6.0; 23–39
Sex, *n* (% Female)	14 (87.5)
Gender Identity, *n* (% Woman)	14 (87.5)
Ethnicity, *n* (% White)	12 (75.0)
Civil Status, *n* (% Single)	8 (50)
Highest Level of Completed Education, *n* (% Post-secondary)	15 (93.8)
Vocational Status, *n* (% Working/Transitioning to Work)	10 (62.5)
Annual Household Income, *n* (% < CAD $100,000)	12 (75.0)
**Medical Characteristics**	
Age at Diagnosis (M years ± SD; range)	27.6 ± 7.9; 15–38
Time Since Diagnosis (M years ± SD; range)	3.2 ± 3.0; 0–10
**Cancer Stage, *n* (%)**	
I	1 (6.3)
II	7 (43.8)
III	3 (18.8)
N/A or “Do Not Know”	5 (31.3)
**Cancer Type, *n* (%)**	
Hematological	4 (25)
Breast	3 (18.8)
Sarcoma	3 (18.8)
Brain	2 (12.5)
Carcinoma	1 (6.3)
Gynecologic	2 (12.5)
Colorectal	0 (0)
Melanoma	1 (6.3)
Testicular	0 (0)
**Treatments Received, *n* (%)**	
Surgery	13 (81.3)
Chemotherapy	11 (68.8)
Radiation	9 (56.3)
Hormonal	3 (18.8)
Other	3 (18.8)
**Perceived Overall Health, *n* (%)**	
Poor to Fair	6 (37.5)
Good to Very Good	10 (62.5)
Excellent	0 (0)
Previous Concussion(s), *n* (%)	2 (12.5)
Cannabis Use in the Past Month, *n* (%)	7 (43.8)

*Notes.* SD = standard deviation.

**Table 3:** Profiles of interviewed participants (*n* = 16).

Participant Pseudonym	Sex	Age	Cancer Stage	Cancer Type	Cancer Treatment	PCI Score	MVPA LSI Score/ Classification
Cole	M	25	II	Hematological	C + R	8	10/Insufficiently active
Emma	F	39	- ^a^	Breast	S + C + R	17	10/Insufficiently active
Erica	F	28	II	Brain	S + C + R	48	25/Active
Eva	F	26	- ^a^	Sarcoma	S + C + R	38	0/Insufficiently active
Ivy	F	32	- ^a^	Brain	S	39	35/Active
Jack	M	26	II	Sarcoma	C + R	54 ^b^	24/Active
Jaime	F	25	- ^a^	Hematological	S + C	31	15/Insufficiently active
Lauren	F	25	- ^a^	Sarcoma	S + C	51	33/Active
Layla	F	29	II	Hematological	C + R	41	24/Active
Mia	F	38	II	Carcinoma	S	35	25/Insufficiently active
Nina	F	36	I	Gynecologic	S	37	5/Insufficiently active
Peyton	F	27	III	Melanoma	S	29	12.5/Insufficiently active
Priya	F	38	III	Gynecologic	S + C	44	10/Active
Sarah	F	39	III	Breast	S + C + R	33	NR
Sydney	F	37	II	Breast	S + C + R	34	46/Active
Taylor	F	23	II	Hematological	S + C	37	10/Insufficiently active

*Notes.* C = chemotherapy; F = female; LSI = Leisure Score Index; M = male; MVPA = moderate-to-vigorous-intensity physical activity (LSI scores ≥ 24 = “active”; LSI scores < 24 = “insufficiently active”); NR = not reported; PCI = perceived cognitive impairment (subscale range: 0–72; scores <54/72 indicate clinically meaningful impairment); R = radiation therapy; S = surgery. ^a^ Reported as “not applicable” or “do not know”. ^b^ Scored on the upper edge of the PCI cut-off value but was invited for an interview to gain male perspective.

## Data Availability

The authors have access to the data in Microsoft Excel and SPSS files. Participants were assured that their data would be kept confidential to the extent permitted by law and that only the research team would have access; thus, the data cannot be shared.

## Supplementary Materials

The following supporting information can be downloaded at: https://www.mdpi.com/article/10.3390/curroncol30060422/s1, File S1: Consolidated Criteria for Reporting Qualitative Research (COREQ) Checklist.
